# Easily identified at-risk patients for extubation failure may benefit from noninvasive ventilation: a prospective before-after study

**DOI:** 10.1186/s13054-016-1228-2

**Published:** 2016-02-26

**Authors:** Arnaud W. Thille, Florence Boissier, Hassen Ben-Ghezala, Keyvan Razazi, Armand Mekontso-Dessap, Christian Brun-Buisson, Laurent Brochard

**Affiliations:** CHU de Poitiers, Réanimation Médicale, Poitiers, France; INSERM CIC 1402 (ALIVE group), Poitiers, France; Université de Poitiers, Faculté de Médecine, Poitiers, France; AP-HP, Hôpital Henri Mondor, DHU A-TVB, Service de Réanimation Médicale, CARMAS Research Group, Créteil, 94010 France; Interdepartmental Division of Critical Care Medicine, University of Toronto, Toronto, ON Canada; Keenan Research Institute and Department of Critical Care Medicine, St. Michael’s Hospital, Toronto, ON Canada

## Abstract

**Background:**

While studies have suggested that prophylactic noninvasive ventilation (NIV) could prevent post-extubation respiratory failure in the intensive care unit, they appear inconsistent with regard to reintubation. We assessed the impact of a prophylactic NIV protocol on reintubation in a large population of at-risk patients.

**Methods:**

Prospective before-after study performed in the medical ICU of a teaching referral hospital. In the control cohort, we determined that patients older than 65 years and those with underlying cardiac or respiratory disease were at high-risk for reintubation. In the interventional cohort, we implemented a protocol using prophylactic NIV in all patients intubated at least 24 h and having one of these risk factors. NIV was immediately applied after planned extubation during at least the first 24 hours. Extubation failure was defined by the need for reintubation within seven days following extubation.

**Results:**

We included 83 patients at high-risk among 132 extubated patients in the control cohort (12-month period) and 150 patients at high-risk among 225 extubated patients in the NIV cohort (18-month period). The reintubation rate was significantly decreased from 28 % in the control cohort (23/83) to 15 % (23/150) in the NIV cohort (p = 0.02 log-rank test), whereas the non-at-risk patients did not significantly differ in the two periods (10.2 % vs. 10.7 %, p = 0.93). After multivariate logistic-regression analysis, the use of prophylactic NIV protocol was independently associated with extubation success.

**Conclusions:**

The implementation of prophylactic NIV after extubation may reduce the reintubation rate in a large population of patients with easily identified risk factors for extubation failure.

## Background

Extubation is a critical decision in an ICU and extubation failure is associated with high mortality [1, 2]. Despite having successfully passed a weaning readiness test, 15 % of patients on average and up to 20–25 % of those at high-risk may need reintubation [3–6].

Several studies have suggested that prophylactic non-invasive ventilation (NIV) could help to prevent post-extubation respiratory failure in patients at high-risk for extubation failure [7–10]. In these studies, NIV was applied during the 24–48 hours following extubation and seemed particularly effective in cases of hypercapnia [7–9] while its use appeared pointless in patients at low-risk for extubation failure [11]. Among the six randomized controlled studies of prophylactic NIV, two studies found a reduction in reintubation rate [7, 10], whereas the others found no significant difference [8, 9, 11, 12]. However, reintubation is the major event independently associated with poor outcome [13], and the main objective of prophylactic NIV should be to avoid reintubation in the most at-risk patients. The studies that have found beneficial effects of NIV in hypercapnic patients included a selected population with chronic pulmonary disease admitted in specialized pulmonary units [7–9]. Consequently, more than 30 % of these patients were hypercapnic at the time of extubation [7, 8], a finding which may be less prevalent in a general ICU.

We previously determined a subset of patients at high-risk for reintubation, comprising patients older than 65 years and/or having underlying cardiac or respiratory disease [5]. Easily identifiable, these criteria represent a large population that could potentially benefit from post-extubation NIV. Therefore, we aimed to assess the risk of reintubation up to seven days after extubation in patients at high-risk for extubation failure after implementation of a specific prophylactic NIV program.

## Methods

### Study design

This is a prospective before-after study aimed at assessing the effects of a protocol for the routine use of prophylactic NIV after planned extubation in patients at high-risk for reintubation. All patients admitted to the 13-bed medical ICU of our teaching hospital in Créteil and who had undergone planned extubation were prospectively screened. In a first cohort treated from May 2005 to May 2006 (control cohort) we identified a population at high-risk for reintubation as noted in our previously published analysis [5]. After implementing prophylactic NIV following extubation, we prospectively collected data from November 2010 to April 2012 to analyze the risk of extubation failure in these patients and compare it to clinicians’ predictions [14]. In the current before-after study we wish to compare the efficacy of this prophylactic NIV protocol. This study was approved by the ethics committee of Henri Mondor hospital (CPP Ile-de-France IX). Signed informed consent was waived because of the observational nature of the study; patients or relatives were nonetheless provided with an informational letter on the aims and conduct of the study.

### Weaning protocol

All ventilated patients were screened every morning by the nurse in charge and a weaning test was systematically performed in those who fulfilled the following weaning criteria: patient awake without continuous infusion of sedatives, SpO_2_ ≥ 90 % with FiO_2_ ≤ 40 % and positive end-expiratory pressure (PEEP) ≤ 5 cmH_2_O, and no need for vasopressors. Failure of the weaning test was defined as the development within 1 hour of any of the following: respiratory rate above 35 breaths/min, SpO_2_ persistently below 90 %, heart rate persistently above 130 beats/min, systolic blood pressure below 90 mmHg or above 180 mmHg, clinical signs suggesting respiratory distress, profuse sweating, agitation or depressed mental status. When the weaning test was well-tolerated, patients were extubated after 1 hour. In the control cohort the weaning test was performed using a T-piece whereas in the NIV cohort it was performed using a pressure-support (PS) around 7 cm H_2_O without PEEP. Moreover, a blood gas test was systematically performed at the end of the weaning test in the NIV cohort.

### Inclusion criteria

In the control cohort, we found that patients older than 65 years and those having any underlying cardiac or respiratory disease were at high-risk for extubation failure with a reintubation rate exceeding 20 % in patients who had one of these two factors, and greater than 30 % when the two factors were combined [5]. Therefore, we decided to implement a protocol for the systematic use of prophylactic NIV within the first 24 hours following planned extubation in all patients who fulfilled at least one of these criteria [14]. Patients intubated less than 24 h and those with a do-not reintubate order were excluded.

Underlying cardiac diseases included left ventricular dysfunction defined by left ventricular ejection fraction ≤ 45 %, ischemic heart disease or chronic atrial fibrillation, history of cardiogenic pulmonary edema, or severe valvulopathy. Chronic lung diseases included chronic obstructive pulmonary disease, obesity-hypoventilation syndrome or restrictive pulmonary disease.

### Prophylactic NIV protocol

The study was conducted after the implementation of a nurse-driven NIV protocol to adjust the ventilatory settings and to improve the patient’s tolerance to NIV following a simple decision algorithm. NIV was immediately applied after extubation for periods of at least 1 hour, with a minimal duration of 8 hours within the first 24 hours following extubation. NIV was initiated using a PS level of 8 cm H_2_O and a PEEP level of 5 cm H_2_O. The nurse could then gradually adjust the PS level to target a tidal volume at around 6-8 ml/kg of predicted body weight. An algorithm was used by nurses in case of leaks, which successively included repositioning of the mask, reducing the PEEP level at 2 cm H_2_O, and reducing the PS level by steps of 2 cm H_2_O. NIV was performed via a non-vented full-face mask with an ICU ventilator using a dedicated NIV mode, equipped with a heated humidifier. Ventilator settings, ventilatory parameters, and blood gases were prospectively collected 1 hour after NIV initiation, as well as the number and duration of NIV sessions. Between NIV sessions patients received standard oxygen therapy; high-flow oxygen therapy was never used, either in the control cohort or in the NIV cohort.

### Duration of NIV and criteria for reintubation

In the absence of acute respiratory failure symptoms 24 hours after planned extubation, NIV was discontinued. If moderate respiratory failure appeared or persisted 24 hours after extubation, prophylactic NIV was continued for periods of 24 hours and reassessed daily until complete disappearance of acute respiratory failure criteria, including: 1) respiratory rate > 25/min, 2) clinical signs suggesting increased work of breathing, 3) respiratory acidosis defined as pH < 7.35 and PaCO_2_ > 45 mmHg, and 4) SpO_2_ < 90 % despite FiO_2_ 0.4.

In the two cohorts, patients were reintubated if they met at least one of the following criteria: 1) clear worsening of respiratory failure with a respiratory rate above 40 breaths per minute, SpO_2_ persistently below 90 % despite supplemental oxygen, worsening pH and PaCO_2_ values with depressed mental status, or persistent inability to remove copious airway secretions; 2) hemodynamic failure (defined as systolic blood pressure < 90 mmHg) without response to a fluid challenge and need for vasoactive drugs, 3) neurological failure defined as altered consciousness, coma or psychomotor agitation, or 4) cardiac or respiratory arrest.

In the literature, the time interval used to define extubation failure varies between 48 [6, 15, 16] and 72 hours [5, 17–19], or up to one week [3, 17, 20, 21]. Since use of NIV may delay reintubation time [3], we defined extubation failure as the need for reintubation within seven days following extubation.

### Statistical analysis

Continuous variables were expressed as mean ± standard deviation and compared using Student’s *t*-test. Dichotomous variables were expressed as percentage and compared using the Chi-2 test. Our main objective was to compare the rate of extubation failure in the two cohorts using the rate of reintubation ≤ 7 days as the primary end point. Kaplan-Meier curves were plotted to assess time from extubation to reintubation in patients at high-risk in the two cohorts and compared by the log-rank test. We performed a multivariate analysis using a backward step-down logistic regression model including the non-collinear variables associated with extubation failure with a p value <0.15 using univariate analysis. We considered two-tailed p values <0.05 as significant.

## Results

**Fig. 1:**
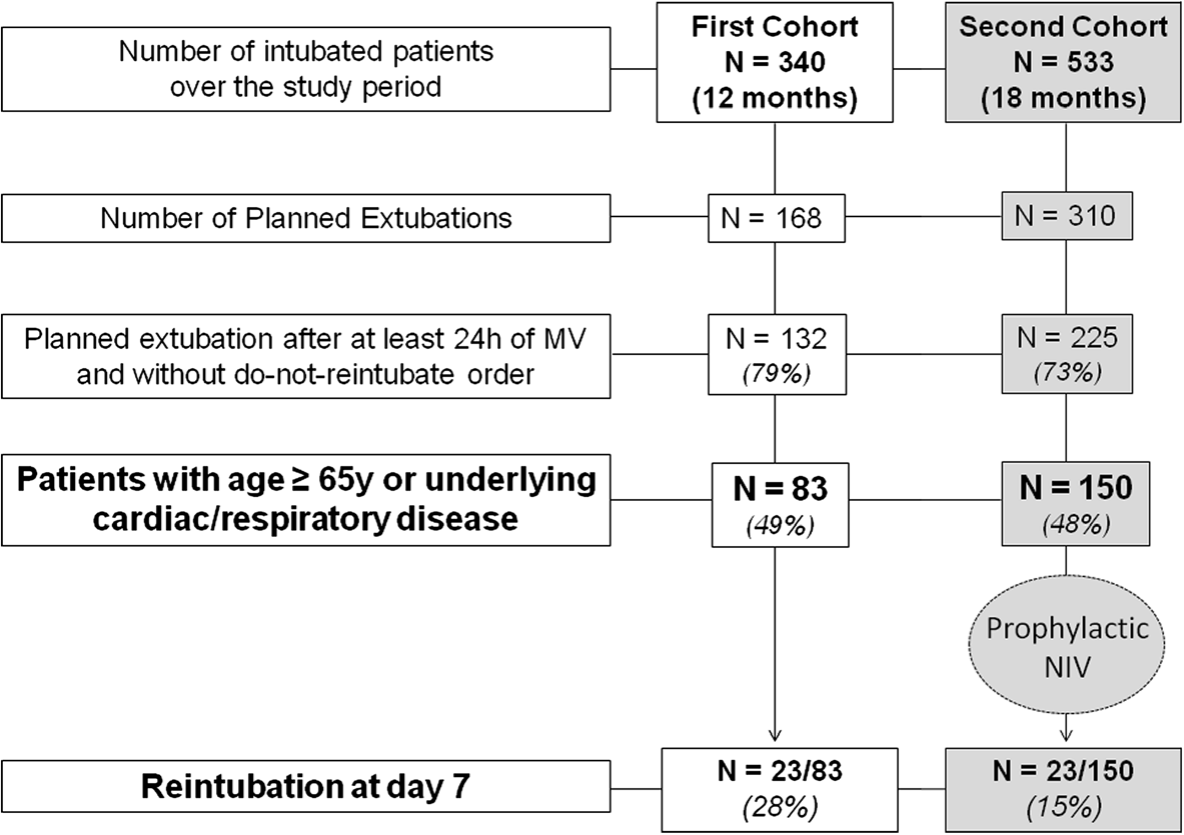
Flow-chart of the study. All in all, 168 patients in the control cohort and 310 in the noninvasive ventilation (NIV) cohort experienced planned extubation. Patients who were extubated after less than 24 h of mechanical ventilation (MV) or with a do-not-intubate order were excluded. Among all patients who experienced planned extubation, the proportion of patients at high-risk for extubation failure was similar in the two cohorts: 49 % (83/168) in the control cohort and 48 % (150/310) in the NIV cohort, p = 0.83

All in all, 132 patients in the control cohort (12-month period) and 225 patients in the NIV cohort (18-month period) experienced planned extubation (Fig. 1). Sixty-one of the 357 extubated patients (17 %) needed reintubation at some time in the ICU, of whom 74 % (n = 45), 82 % (n = 50) and 97 % (n = 59) were reintubated within the first 48 hours, 72 hours and seven days following extubation, respectively. Acute respiratory failure was the main reason for reintubation (69 %, 42/61). The mortality of patients who needed reintubation reached 51 % (31/61).

### Patients at high-risk for extubation failure

In the control cohort, 83 of the 132 extubated patients (63 %) were at high-risk for extubation failure (Fig. 1), and none of them received any prophylactic NIV. In the NIV cohort, 150 of the 225 extubated patients (67 %) were at high-risk and eligible to receive prophylactic NIV. Patients’ characteristics did not differ between the two cohorts (Table 1).

**Table 1:** Comparison of the patients at high-risk for reintubation in the 2 cohorts

	Control cohort N = 83	NIV cohort N = 150	P value
Patients’ characteristics			
Age, years	66 ± 15	69 ± 11	0.05
Age ≥ 65 years, n (%)	54 (65 %)	109 (73 %)	0.23
Male sex, n (%)	49 (59 %)	85 (57 %)	0.73
SAPS II at admission, points	50 ± 19	49 ± 18	0.44
Underlying cardiac disease, n (%)	45 (54 %)	90 (60 %)	0.39
Underlying chronic lung disease, n (%)	31 (37 %)	53 (35 %)	0.76
Reason for intubation			0.81
Acute respiratory failure, n (%)	33 (39 %)	64 (43 %)	
Shock, n (%)	14 (17 %)	29 (19 %)	
Coma or neurologic disorders, n (%)	13 (16 %)	25 (17 %)	
Postoperative, n (%)	20 (24 %)	26 (17 %)	
Cardiac arrest, n (%)	3 (4 %)	6 (4 %)	
Variables at time of extubation			
SOFA at time of extubation, points	3.4 ± 2.2	3.6 ± 2.3	0.56
PaO_2_/FiO_2_ ratio, mm Hg	283 ± 95	296 ± 92	0.30
pH, units	7.46 ± 0.06	7.45 ± 0.05	0.73
PaCO_2_, mm Hg	41 ± 10	39 ± 8	0.15
PaCO_2_ > 45 mm Hg, n (%)	24 (29 %)	27 (18 %)	0.06
Duration of MV prior to extubation, days	8.0 [5.0–13.8]	6.0 [4.0–11.8]	0.24
Outcome			
Reintubation < 48 h, n (%)	18 (22 %)	16 (11 %)	0.03
Reintubation < 72 h, n (%)	20 (24 %)	19 (13 %)	0.02
Reintubation < 7 days, n (%)	23 (28 %)	23 (15 %)	0.02
Reintubation at any time in ICU, n (%)	24 (29 %)	24 (16 %)	0.02
Total duration of invasive MV, days	9.0 [5.0–16.0]	7.0 [4.0–13.8]	0.07
ICU length of stay, days	14 [11.0–22.0]	12.0 [7.0–20.0]	0.18
In-ICU mortality, n (%)	12 (14 %)	16 (11 %)	0.39

Values are given as mean ± standard deviation (SD) or as median [interquartile range, from 25^th^ to 75^th^ percentiles]

*NIV* noninvasive positive pressure ventilation, *SAPS II* Simplified Acute Physiology Score II, *SOFA* Sequential Organ Failure Assessment, *MV* mechanical ventilation, *ICU* intensive care unit

**Fig. 2:**
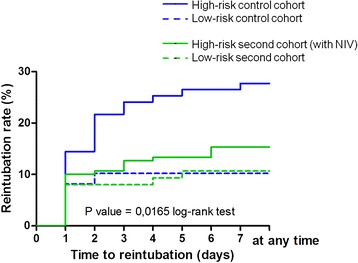
The Kaplan-Meier plots of the cumulative reintubation rates within seven days following extubation. The rate of extubation failure (reintubation at day 7) significantly differed between the four groups (p = 0.0165 log-rank test). Among the patients at high-risk, this rate was significantly lower in the NIV cohort (*green solid line*) than in the control cohort (*blue solid line*), decreasing from 28 % (23/83) to 15 % (23/150), p = 0.0225 by log-rank test. The difference remained significantly lower after having excluded the 11 patients in the NIV cohort who did not effectively receive NIV: 17 % (23/139) vs. 28 % (23/89), p = 0.0466 log-rank test. By contrast, the rate of extubation failure did not significantly differ during the two periods in patients at low-risk: 10.2 % (5/49) in the control cohort (*blue dotted line*) vs. 10.7 % (8/75) in the NIV cohort (*green dotted line*), p = 0.93

All in all, reintubation within seven days following extubation occurred in 46 of the 233 patients considered at high-risk (20 %). This rate was significantly lower in the NIV cohort than in the control cohort, decreasing from 28 % (23/83) to 15 % (23/150), p = 0.02 log-rank test (Fig. 2) whereas the non-at-risk patients did not significantly differ during the two periods: 10.2 % (5/49) vs. 10.7 % (8/75), p = 0.93. The rate of reintubation in patients at high-risk treated with NIV was not significantly higher than that in patients at low-risk overall: 15.3 % (23/150) vs. 10.5 % (13/124), p = 0.24.

### Use of NIV

Among the 150 patients considered at high-risk for reintubation in the NIV cohort, 139 (93 %) actually received prophylactic NIV after extubation. In the 11 remaining patients the prophylactic NIV protocol was not followed by the physician or refused by the patient (these patients were kept in the analysis of the NIV cohort performed on an intention-to-treat basis). Given the fact that none of these 11 patients needed reintubation, the rate of extubation failure reached 17 % (23/139) among all patients treated with NIV but remained significantly lower than in patients at high-risk in the control cohort (p = 0.04 log-rank test).

The median duration of NIV within the first 24 h following extubation was 490 min [interquartile range, 353-559] and 54 % of the patients (75/139) received more than eight hours of NIV. NIV was prolonged beyond the first 24 hours in 24 % of the cases (33/139) because of persistent respiratory failure with a rate of extubation failure of 21 % (7/33).

### Factors associated with extubation failure in high-risk patients

A comparison of patients in cases of success or failure of extubation is given in Table 2. After adjustment using multivariate logistic-regression, patients with a more prolonged duration of mechanical ventilation prior to extubation were more likely to experience extubation failure, whereas the implementation of a prophylactic NIV protocol was independently associated with extubation success (Table 3).

**Table 2:** Variables associated with reintubation within the 7 days following extubation among all patients at high-risk for extubation failure

	Extubation Success, N = 187	Reintubation at day 7, N = 46	P value
Patients’ characteristics			
Age, years	68 ± 14	69 ± 10	0.46
Age ≥ 65 years, n (%)	128 (68 %)	35 (76 %)	0.31
Male sex, n (%)	103 (55 %)	31 (67 %)	0.13
SAPS II at admission, points	48 ± 18	53 ± 19	0.13
Underlying cardiac disease, n (%)	107 (57 %)	28 (60 %)	0.65
Underlying chronic lung disease, n (%)	69 (37 %)	15 (33 %)	0.59
Use of prophylactic NIV protocol	127 (68 %)	23 (50 %)	0.02
Reason for intubation			0.40
Acute respiratory failure, n (%)	78 (42 %)	19 (41 %)	
Shock, n (%)	38 (20 %)	5 (11 %)	
Coma or neurologic disorders, n (%)	31 (17 %)	7 (15 %)	
Postoperative, n (%)	34 (18 %)	12 (26 %)	
Cardiac arrest, n (%)	6 (3 %)	3 (7 %)	
Variables at time of extubation			
SOFA at time of extubation, points	3.4 ± 2.2	3.7 ± 2.7	0.46
PaO_2_/FiO_2_ ratio, mm Hg	289 ± 90	300 ± 103	0.47
PaO_2_/FiO_2_ ratio ≤ 200 mm Hg, n (%)	30 (16 %)	6 (13 %)	0.61
pH, units	7.45 ± 0.06	7.46 ± 0.06	0.63
PaCO_2_, mm Hg	41 ± 9	38 ± 8	0.16
PaCO_2_ > 45 mm Hg, n (%)	42 (23 %)	9 (20 %)	0.67
Duration of MV prior to extubation, days	6.0 [4.0–11.0]	13.0 [5.0–20.0]	<0.001
Duration of MV prior to extubation > 7 days, n (%)	77 (41 %)	29 (63 %)	<0.01

Values are given as mean ± standard deviation (SD) or as median [interquartile range, from 25th to 75th percentiles]

*SAPS II* Simplified Acute Physiology Score II, *NIV* noninvasive positive pressure ventilation, SOFA Sequential Organ Failure Assessment, *MV* mechanical ventilation, *ICU* intensive care unit

**Table 3:** Variables independently associated with reintubation within the 7 days following extubation in patients at high-risk for extubation failure (N = 233)

Multivariate analysis using logistic regression	Adjusted Odds Ratio^a^ [95 % CI]	P value
Duration of MV prior to extubation, per day	1.09 [1.05–1.13]	<0.001
Use of prophylactic NIV protocol	0.48 [0.24–0.96]	0.04

All variables significantly associated with extubation failure with a p value < 0.15 were included in the model including: male sex, SAPS II, hypercapnia, duration of mechanical ventilation (MV) prior to extubation, and application of prophylactic NIV protocol (NIV cohort)

*SAPS II* Simplified Acute Physiology Score II, *NIV* noninvasive positive pressure ventilation

^a^Values of adjusted odds ratio are taken from the final model including only variables independently associated with extubation failure. Logistic regression was performed using 233 observations and the final model had an area under a Receiver Operating Curve of 0.726 (Hosmer-Lemeshow test 0.683)

In the NIV cohort, the proportion of hypercapnic patients (PaCO_2_ > 45 mmHg) at the end of the weaning test was 16 % overall (35/225) and 20 % among the patients at high-risk (30/150).

## Discussion

In this prospective before-after study we assessed the impact of NIV on patient outcome after planned extubation. The implementation of a prophylactic NIV protocol significantly reduced the rate of reintubation in patients identified as at high-risk of extubation failure. Our NIV protocol was applied in a large population of patients at high-risk based on particularly easily identifiable criteria, including those older than 65 years or with underlying cardiac or respiratory disease.

Several studies have suggested that prophylactic NIV could reduce the risk of post-extubation respiratory failure, particularly in hypercapnic patients [7–9]. In these studies, the rate of reintubation assessed within the 48–72 hours following extubation ranged from 19 to 24 % with standard oxygen and from 8 to 11 % with prophylactic NIV [7–9]. While reintubation rates were higher in our population, we defined extubation failure as reintubation up to seven days after extubation. When assessed within the first 48 hours following extubation, our reintubation rates are similar to those reported in previous studies (22 % in the control cohort and 11 % in the NIV cohort).

To date, only two studies have found a significant reduction in the rate of reintubation [7, 10]. In the study by Nava and colleagues, prophylactic NIV applied at least eight hours per day during the first 48 hours following extubation significantly reduced the rate of reintubation from 24 % to 8 % (p = 0.027). Another study observed a decrease in reintubation rate from 39 % to only 5 % in the group receiving NIV (p = 0.016) [10]. However, only 38 patients were included in this single-center study and the rate of reintubation reported in the control group receiving standard oxygen (39 %) was inordinately high [10].

In the study by Ferrer and colleagues, prophylactic NIV helped to reduce the risk of post-extubation respiratory failure and to decrease mortality, even though the reintubation rate was not significantly decreased [8]. Indeed, as NIV could be used as rescue therapy in case of respiratory distress, it enabled a number of patients from the control group receiving standard oxygen to avoid reintubation. As NIV was chiefly beneficial in hypercapnic patients with chronic respiratory disorders, the same group conducted a second trial including 106 hypercapnic patients [9]. The results confirmed those of the previous study, and use of prophylactic NIV avoided post-extubation respiratory failure [9]. Although 90-day mortality was significantly lower in patients receiving prophylactic NIV, neither in-ICU mortality nor in-hospital mortality differed between the two groups, and it would perhaps be premature to attribute the difference in long-term outcome to the use of NIV immediately after extubation. In our study, although we found a significant decrease in the reintubation rate (13 %), ICU mortality did not differ between patients treated with NIV and those receiving standard oxygen.

Whereas NIV seems beneficial in patients considered at high-risk for reintubation, it is probably pointless in patients at low-risk. In a large multicenter trial including nearly 400 patients intubated for more than 48 hours, the use of prophylactic NIV had no impact on outcome [11]. The patients included in this study were not really at high-risk for reintubation, and the overall reintubation rate did not exceed 10 %.

In studies including patients considered at high-risk for extubation failure [7–10], the inclusion criteria were highly heterogeneous and differed from one study to another. Nava and colleagues included patients with hypercapnia (34 % of the patients) as well as patients with chronic cardiac failure, several comorbidities, weak cough, stridor, or having failed several weaning tests before extubation [7]. Ferrer and colleagues included patients aged ≥ 65 years or more, a high severity score, or intubated for cardiac failure [8]. The same group subsequently conducted a second trial including only patients with hypercapnia [9]. In another study, prophylactic NIV was used only in patients intubated at least three days for acute respiratory failure [10]. However, in these studies many of the factors constituting inclusion criteria to start prophylactic NIV are not variables clearly associated with reintubation.

From our standpoint, future research efforts should focus on identifying patients at high-risk for reintubation who may benefit from NIV in various ICU settings. On the other hand, classification of patients according to the difficulty of their weaning process [22] seems of little help in prediction of extubation failure since the reintubation rate between patients with simple weaning and those with difficult weaning is somewhat similar [4, 23–25]. By contrast, we have found that prolonged duration of mechanical ventilation prior to extubation was a strong predictor of extubation failure, and that patients intubated more than seven days may probably be considered as at high-risk of extubation failure [14]. Since our first study [5], we decided to initiate NIV in a large subset of patients, who were at once particularly easy to identify, and at unacceptably high-risk for reintubation and mortality. Among these patients, only 20 % had hypercapnia during the weaning test and would have received prophylactic NIV in the event that only this criterion had been used.

### Limitations

First, our study was performed in a single center in which our population may have differed from those of other centers in terms of demographic or primary reason for intubation, a factor that limits the generalizability of our results. Although cohort studies obviously do not have the strength of a randomized controlled study, our study was prospective and included the largest number of patients at high-risk treated with prophylactic NIV to date (n = 139). The reintubation rate in the non-at-risk patients remained exactly similar between the two periods.

Second, the weaning readiness tests were not performed in the same way in the two cohorts. Low levels of PS were used during the NIV cohort, which may have resulted in lower levels of work of breathing than with T-piece as performed in the first cohort [26]. However, clinical studies have found no difference between the two approaches in terms of extubation outcome [15] and, if anything, the low pressure-support approach could result in a higher extubation rate and potentially more patients in need of reintubation in the NIV cohort.

Third, the data from the NIV cohort were collected four years after the end of the control cohort. Therefore, other changes may have impacted the reintubation rate, such as modification in sedation practice, use of diuretics, special attention paid to patients at high-risk, and our result could be due to an improvement in the process of weaning including prophylactic NIV rather than the implementation of NIV alone. However, the duration of mechanical ventilation prior to extubation was not significantly different between cohorts and the use of the NIV protocol remained independently associated with extubation success, even after adjustment on prior duration of mechanical ventilation. Moreover, the percentage of patients at high-risk was not significantly different between cohorts, thereby suggesting a relatively similar clinical practice.

As a final limitation, the trend toward a lower proportion of hypercapnic patients in the NIV cohort may have partially contributed to the lower reintubation rate compared to the control cohort. However, although it was previously shown that weaning may be more prolonged in hypercapnic patients, it was not significantly associated with extubation failure [4]. In our second cohort, hypercapnia at the end of the weaning test was associated with extubation failure in univariate analysis, but was not an independent predictor of reintubation after adjustment on multiple risk factors [14].

Although our results support the use of NIV rather than standard oxygen in patients at-risk, the beneficial effects found in this study might not be extrapolated to patients treated by high-flow oxygen therapy [27].

## Conclusions

Prophylactic NIV applied immediately after extubation could reduce the reintubation rate in a large population of patients at high-risk for extubation failure including those aged 65 years or older and/or having underlying cardiac or respiratory disease.

## Key messages

- The implementation of a protocol using prophylactic noninvasive ventilation immediately after extubation may reduce the reintubation rate in a large population of patients with easily identified risk factors for extubation failure.
- These patients were older than 65 years and presented with underlying cardiac or respiratory disease.
